# Assessing the reaction to and efficacy of the Screener drug discovery and development board game as a pedagogical tool in postgraduate courses

**DOI:** 10.1590/1414-431X2023e13258

**Published:** 2024-01-22

**Authors:** F. Noël, G. Xexéo, M.A. Martins, E.J.R. Silva, A.S. Pupo, P.J.C. Magalhães, R.C.P. Lima-Júnior, K.K.L. Gadelha, K. Lima-Silva, J.M. Raimundo, P.C. Ghedini, M.E. Crespo-Lopez, G.P. Arrifano, J. Ferreira, R.D. Prediger, G.C.G. Militão, R.B. Oliveira, A.W. Hollais, L.C.M. Rodrigues, D.T. Carvalho, S.K.P. Costa, D.T.O. Martins

**Affiliations:** 1 Programa de Pós-Graduação em Farmacologia e Química Medicinal, Universidade Federal do Rio de Janeiro, Rio de Janeiro, RJ, Brasil; 2 Laboratório de Ludologia, Engenharia e Simulação, Programa de Engenharia de Sistemas e Computação, COPPE, Universidade Federal do Rio de Janeiro, Rio de Janeiro, RJ, Brasil; 3 Laboratório de Inflamação, Instituto Oswaldo Cruz, Fundação Oswaldo Cruz, Rio de Janeiro, RJ, Brasil; 4 Departamento de Biofísica e Farmacologia, Instituto de Biociências de Botucatu, Universidade Estadual Paulista, Botucatu, SP, Brasil; 5 Programa de Pós-Graduação em Farmacologia, Departamento de Fisiologia e Farmacologia, Faculdade de Medicina, Universidade Federal do Ceará, Fortaleza, CE, Brasil; 6 Grupo de Pesquisa em Farmacologia de Produtos Bioativos, Centro Multidisciplinar UFRJ-Macaé, Universidade Federal do Rio de Janeiro, Macaé, RJ, Brasil; 7 Departamento de Farmacologia, Instituto de Ciências Biológicas, Universidade Federal de Goiás, Goiânia, GO, Brasil; 8 Laboratório de Farmacologia Molecular, Instituto de Ciências Biológicas, Universidade Federal do Pará, Belém, PA, Brasil; 9 Programa de Pós-graduação em Farmacologia, Centro de Ciências Biológicas, Universidade Federal de Santa Catarina, Florianópolis, SC, Brasil; 10 Departamento de Fisiologia e Farmacologia, Centro de Biociências, Universidade Federal de Pernambuco, Recife, PE, Brasil; 11 Programa de Pós-Graduação em Ciências Farmacêuticas, Faculdade de Farmácia, Universidade Federal de Minas Gerais, Belo Horizonte, MG, Brasil; 12 Programa de Pós-Graduação em Ciências Fisiológicas, Universidade Federal do Espírito Santo, Vitória, ES, Brasil; 13 Faculdade de Ciências Farmacêuticas, Universidade Federal de Alfenas, Alfenas, MG, Brasil; 14 Departamento de Farmacologia, Instituto de Ciências Biomédicas, Universidade de São Paulo, São Paulo, SP, Brasil; 15 Programa de Pós-Graduação em Ciências da Saúde, Laboratório de Farmacologia, Faculdade de Medicina, Universidade Federal de Mato Grosso, Cuiabá, MT, Brasil

**Keywords:** Game-based learning, Educational game, Survey, Drug discovery, Postgraduate course, Active learning

## Abstract

Screener, a board game supplemented with online resources, was introduced and distributed by the Brazilian Society of Pharmacology and Experimental Therapeutics to postgraduate programs as an instructional tool for the process of drug discovery and development (DDD). In this study, we provided a comprehensive analysis of five critical aspects for evaluating the quality of educational games, namely: 1) description of the intervention; 2) underlying pedagogical theory; 3) identification of local educational gaps; 4) impact on diverse stakeholders; and 5) elucidation of iterative quality enhancement processes. We also present qualitative and quantitative assessments of the effectiveness of this game in 11 postgraduate courses. We employed the MEEGA+ online survey, comprising thirty-three close-ended unipolar items with 5-point Likert-type response scales, to assess student perceptions of the quality and utility of Screener. Based on 115 responses, the results indicated a highly positive outlook among students. In addition, we performed a preliminary evaluation of learning outcomes in two courses involving 28 students. Pre- and post-quizzes were applied, each consisting of 20 True/False questions directly aligned with the game's content. The analysis revealed significant improvement in students' performance following engagement with the game, with scores rising from 8.4 to 13.3 (P<0.0001, paired *t*-test) and 9.7 to 12.7 (P<0.0001, paired *t*-test). These findings underscore the utility of Screener as an enjoyable and effective tool for facilitating a positive learning experience in the DDD process. Notably, the game can also reduce the educational disparities across different regions of our continental country.

## Introduction

Over the last ten years (2012-2022), the number of research papers using the term game-based learning has dramatically increased from 381 to 6,765 results, according to a SCOPUS research. The use of games as a strategy for active learning has been applied in all areas of healthcare (1), including pharmacy, as recently reviewed (2,3) and specific disciplines, such as pharmacology (4). This phenomenon can be explained by the fact that the use of games or game elements improve student engagement and that learning is optimized by gaming mechanisms, such as immediate feedback, active participation, repeated practice, motivation, and teamwork (2,3). Although game elements are widely recognized as important for enhancing student motivation and learning outcomes, the underlying mechanisms are not yet well-understood. However, recent findings obtained through infrared spectroscopy provide some direct evidence of increased brain activation in areas associated with emotion and reward processing in adults who played a game-based version of a task (5). The authors also reported activation of frontal areas associated with attention.

As part of multiple game-based learning strategies, board games are becoming increasingly popular in various fields, including medicine (6). On the other hand, such games are scarce for the drug discovery and development (DDD) process and have various limitations, as reported in Supplementary Table S1.

Based on this observation, we created and recently launched Screener (7), a board game with online resources, endorsed and distributed by the Brazilian Society of Pharmacology and Experimental Therapeutics (SBFTE). Screener can also be freely printed at home from https://www.screener.com.br/.

We designed this game with a primary target audience in mind: students pursuing Master's and/or PhD degrees in Pharmacology, Medicinal Chemistry, Toxicology, and Pharmacy. The aim was to help students learn about the main stages of the DDD process and the different kinds of assays that need to be performed. Furthermore, we wanted them to appreciate that DDD is a collaborative effort of experts with diverse skills (7). Another objective was for the game to serve as a tool for the creation of new disciplines on DDD and to strengthen the network of postgraduate courses in pharmacology, as part of the educational initiatives of SBFTE.

The present work aimed to assess the efficacy of the final version of the Screener board game as a pedagogical tool in eleven postgraduate courses (June 2022-June 2023) in different regions of Brazil. Present results support the hypothesis that Screener is useful as an active learning tool for teaching the complex process of DDD to postgraduate students.

## Material and Methods

The SQUIRE-EDU (Standards for Quality Improvement Reporting Excellence in Education) guidelines (8) were selected to guide the present report on the use of Screener. Based on these guidelines, we described in detail the following five topics considered important for the quality assessment of educational games (1): description of the intervention, guiding theory, identification of the local gap, impact on broad stakeholders, and explanation of iterative quality improvement.

### Intervention description

The game was fully described in our previous paper (7), including its components, dynamics, and rules. In summary, the game involves up to six players or teams representing companies that are competing to register a new drug product. To achieve this goal, players must complete all seven stages of the DDD process. Collaboration among players is key, as they work together to collect a set of 29 task cards, which must be purchased in the correct order. These task cards are divided into four categories: efficacy, safety, pharmacokinetics, and pharmaceutical development. Each card features a QR code that players can scan to access explanatory text. Players also receive power cards and banknotes to facilitate the purchase of task cards and the payment of possible setbacks, which are represented by bonus/setback cards that arise during the game. Due to local constraints, the usage of the game varied, as shown in Table 1. In six courses, Screener served as the central component of a dedicated DDD discipline, while in other courses, it was integrated into existing disciplines with a broader scope, such as Pharmacology or Medicinal Chemistry. Consequently, the duration of gameplay ranged from 5 to 11 h (spanning 2-5 days) to 2-3 h (usually completed in a single session).

**Table 1:** Postgraduate courses that used the SCREENER game in the classroom and description of game usage and participants: undergraduate research students (UG), Master's (MA) and Doctoral (PhD) students, and Post-doctoral (PD) fellows.

Institution and course	Game use and discipline type Sessions (playing time)	Survey responses(total)	Student levelsUG / MA / PhD / PD
UFRJ* Pharmacology and Medicinal Chemistry	New, DDD-centered 3 days (4-5 h)	16 (16)	0 / 10 / 6 / 0
UFRJ-Macaé Bioactive products and Biosciences	New, DDD-centered 2 days (5 h)	13 (13)	4 / 7 / 2 / 0
UFG Biological Sciences	New, DDD-centered 4 days (10 h)	12 (12)	1 / 3 / 8 / 0
UFPA Pharmacology and Biochemistry	New, DDD-centered 5 days (11 h)	9 (9)	0 / 4 / 5 / 0
UFSC* Pharmacology	New, DDD-centered 4 days (9 h)	8 (12)	0 / 3 / 5 / 0
UFPE Biochemistry and Physiology	Old, not DDD-centered 1 day (2.5 h)	15 (17)	0 / 11 / 4 / 0
UFMG Pharmaceutical Sciences	Old, not DDD-centered 2 days (3 h)	5 (5)	2 / 1 / 2 / 0
UFES Physiological Sciences	Old, not DDD-centered 1 day (3 h)	4 (5)	0 / 3 / 1 / 0
UFC** Pharmacology	Old, not DDD-centered 1 day (2.75 h)	20 (24)	1 / 14 / 5 / 0
UNESP Biomolecular and Pharmacological Sciences	New, DDD-centered 3 days (8 h)	9 (9)	0 / 5 / 2 / 2
UNIFAL Pharmaceutical Sciences	Old, not DDD-centered 1 day (2 h)	3 (3)	0 / 2 / 1 / 0

*Courses offered the discipline twice, and the students' answers to the survey were grouped. **Students were divided into two sessions. DDD: drug discovery and development; UFRJ: Federal University of Rio de Janeiro; UFG: Federal University of Goiás; UFPA: Federal University of Pará; UFSC: Federal University of Santa Catarina; UFPE: Federal University of Pernambuco; UFMG: Federal University of Minas Gerais; UFES: Federal University of Espírito Santo; UFC: Federal University of Ceará; UNESP: São Paulo State University “Júlio de Mesquita Filho”; UNIFAL: Federal University of Alfenas.

### Guiding theory

Prensky's assertions from over two decades ago (9) have sparked ongoing discussions about how students have evolved and no longer fit the mold of those whom the education system was originally designed to teach: today's learners are motivated by instant gratification and frequent rewards and tend to prefer games to traditional forms of serious work. Lectures, step-by-step logic, and “tell-test” instruction methods are less appealing to present-day students, and they prefer active learning (9). As a result, serious games are effective tools that can boost student engagement (1-3). In this gaming culture, board games occupy a vital space in education for 21st century society (10).

### Local gap

A lack of educational programs and qualified professionals who are well-versed in all phases of the DDD process has been identified as a barrier to Brazil's advancement in providing innovative drugs (11). Given that many undergraduate and postgraduate students in Brazil have difficulties with the English language, we recognized the importance of creating an educational game in Portuguese to ensure that language barriers do not hinder the game’s dissemination. Additionally, even postgraduate students who work on certain aspects of drug discovery within academia have a limited perspective on the complexity of the DDD process and the multitude of assays that must be performed before registering a new drug product with regulatory agencies.

### Impact on stakeholders

#### Student perception

We used the MEEGA+ questionnaire to assess the quality and usefulness of the game from the students' perspective. This online survey was completed anonymously by the students and consisted of thirty-three close-ended one-sided items with 5-point Likert-type answer options (12,13). The questionnaire began with an Informed Consent Form, informing that the procedures of this research strictly adhered to the regulations indicated by the Brazilian General Data Protection Law (No. 13.709/2018).

#### Assessment of learning

To assess this dimension, we conducted studies at two of the postgraduate courses (UFC and UNESP-Botucatu; Table 1) in June 2023 to explore how effectively the specific content of the DDD process had been learned. To achieve this, we used pre- and post-quizzes. We administered the pre-quiz just before starting the game, without informing the students that there would be a post-quiz, and explaining that the pre-quiz had no effect on their score in the discipline. Following a guide for this evaluation tool (https://www.europlanet-society.org/outreach/europlanet-evaluation-toolkit/evaluation-tool-pre-post-quizzes/), the pre- and post-quiz questions were identical, with only the order of questions changed. The post-quiz was administered immediately after the game had ended or the day after. The quiz consisted of 20 true/false questions directly related to the content covered by the game. To minimize the weight of guessed answers, we used a scoring system where ‘1' was given to a correct answer if the “For sure” option was selected, ‘0.5' was given to a correct answer if the ‘Not sure' option was selected, and ‘0' was given for an incorrect answer. The students were unaware of the scoring strategy.

#### Iterative quality improvement

Our team developed the game during the COVID-19 pandemic and tested the rules on a weekly basis through online meetings, using the “Tabletop Simulator” platform to comply with social distancing measures. To avoid repeating information and shorten the excessively long game time, we made significant changes to the original competition-style game, transforming it into a semi-cooperative game where all players work together to collect a single set of cards.

In a second round of quality assessment, a black-and-white draft version of Screener was tested with six students participating in a regular discipline on DDD in the postgraduate program in Pharmacology and Medicinal Chemistry of the Federal University of Rio de Janeiro. The students provided feedback, and several of their suggestions were incorporated into the final version of the game. These included adding a physical grid to delimit the card spaces, positioning the initial card at the center of the board instead of a corner, providing small tokens to indicate the total number of cards acquired during the game, and including suggestions in the rule book to help consolidate knowledge. Additionally, tips were added to the rule book to make it easier to save the game situation at the end of a match and resume gameplay on the day of the next match.

## Results

### Quantitative assessment of the use of Screener in postgraduate courses

#### Student perception

**Figure 1:**
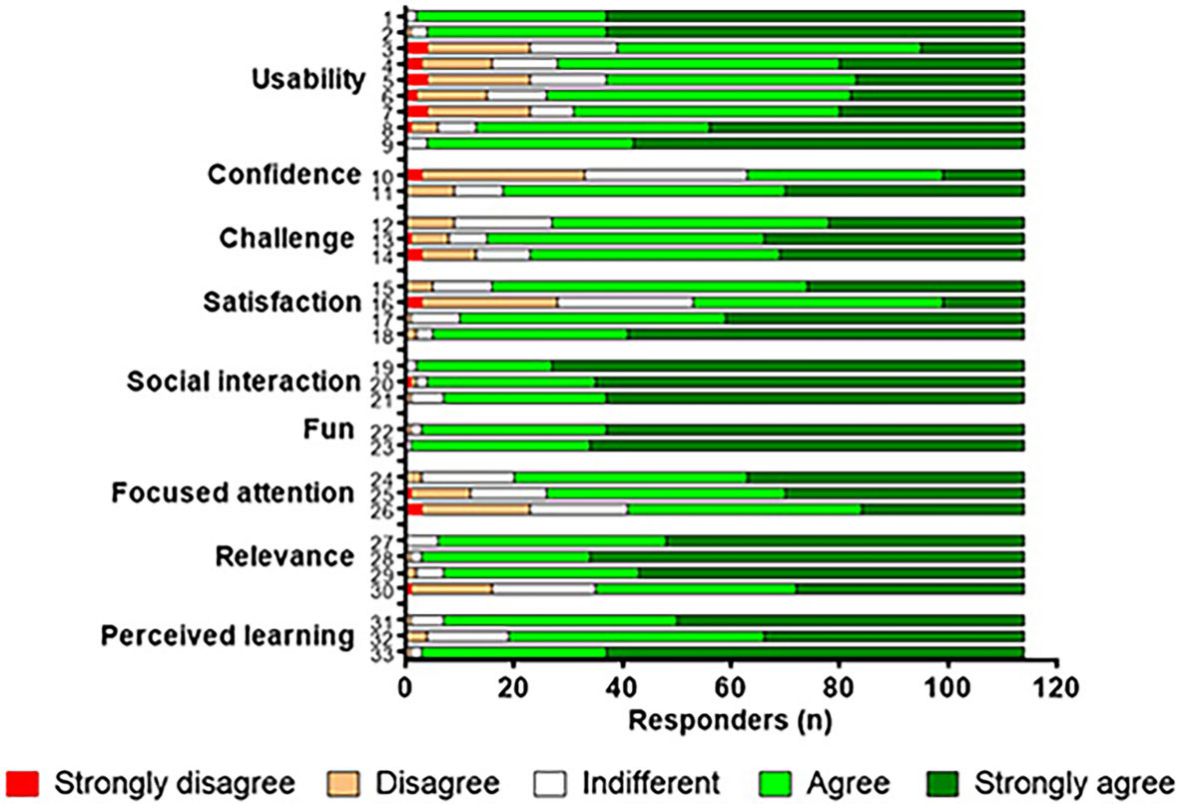
Result of students' opinion survey. Students voluntarily and anonymously reported their level of agreement to 33 items according to the MEEGA+ questionnaire. Answers are based on a 5-point Likert-type scale and data are reported in number of responders (115 players from 11 postgraduate courses - see list in Table 1). The items of the questionnaire cover: USABILITY: 1) The game design is attractive (interface, graphics, cards, boards, etc.). 2) The font and colors are well blended and consistent. 3) I needed to learn a few things before I could play the game. 4) Learning to play this game was easy for me. 5) I think that most people would learn to play this game very quickly. 6) I think that the game is easy to play. 7) The game rules are clear and easy to understand. 8) The fonts (size and style) used in the game are easy to read. 9) The colors used in the game are meaningful. CONFIDENCE: 10) When I first looked at the game, I had the impression that it would be easy for me. 11) The contents and structure helped me become confident that I would learn with this game. CHALLENGE: 12) This game is appropriately challenging for me. 13) The game provides new challenges at an appropriate pace. 14) The game does not become monotonous as it progresses (repetitive or boring tasks). SATISFACTION: 15) Completing the game tasks gave me a satisfying feeling of accomplishment. 16) It was due to my personal effort that I managed to advance in the game. 17) I feel satisfied with what I learned from the game. 18) I would recommend this game to my colleagues. SOCIAL INTERACTION: 19) I was able to interact with other players during the game. 20) The game promotes cooperation and/or competition among the players. 21) I felt good interacting with other players during the game. FUN: 22) I had fun with the game. 23) Something happened during the game that made me smile. FOCUSED ATTENTION: 24) There was something interesting at the beginning of the game that captured my attention. 25) I was so involved in my gaming task that I lost track of time. 26) I forgot about my immediate surroundings while playing this game. RELEVANCE: 27) The game content is relevant to my interests. 28) It is clear to me how the content of the game relates to the course. 29) This game is an adequate teaching method for this course. 30) I prefer learning with this game to learning through other ways. PERCEIVED LEARNING: 31) The game contributed to my learning in this course. 32) The game allowed for efficient learning compared with other activities in the course. 33) The game contributed to learning the concepts about the topic of drug discovery and development process.

A total of 115 students responded to the MEEGA+ questionnaire, which was highly representative of the 124 players (mainly Master's and PhD students) who used the game in one of the eleven postgraduate courses. Overall, the survey results indicated that Screener was well accepted, as reflected by the large majority of “agree” and “strongly agree” responses (See green lines in Figure 1). The game was particularly well-rated in terms of social interaction, fun, relevance, and perceived learning, as indicated by a majority of “strongly agree” responses for these dimensions. Notably, the students also reported that they had a sense of achievement and overall satisfaction at the end of the game. More than 85% answered “agree” or “fully agree” in response to the following statements within the “Satisfaction” dimension: “Completing the game tasks gave me a satisfying feeling of accomplishment”, “I am satisfied with what I learned from the game,” and “I would recommend this game to my colleagues” (see Figure 1, items 15, 17, and 18).

Despite the generally positive evaluations, some items received a lower score. Specifically, items related to the required prior knowledge, the ease of play, and the clarity of rules were evaluated positively, but to a lesser extent (see Figure 1, items 3-7 of the “Usability” dimension and item 10 of the “Confidence” dimension). Another group of items about perceived lack of challenge and monotony (items 13 and 14 of the “Challenge” dimension) and the feeling that personal effort was not particularly crucial (item 16 of the “Satisfaction” dimension) also received lower scores, which was expected due to the collaborative nature of this game. Although we previously decided not to include the quantitative data from a course (ICB, USP) that used the game in a hybrid form (some students in the classroom and most of them online), the data were not very different from those presented in Figure 1.

#### Assessment of learning

As a first step towards achieving learning assessment, we developed a true or false quiz comprising 20 questions based on the game's content (see Methods). The quiz was administered to undergraduate Pharmacy students during their first pharmacology class in the fifth semester. The objective was to assess whether the quiz could identify the level of technical knowledge regarding the DDD process. Out of the 58 students who completed the quiz, the average score was 7.2±2.0 (SD) out of 20, which is 36% of the maximum possible score. This score falls below the expected score for random guessing (50%), suggesting that the quiz was potentially effective in detecting improvements in learning about the DDD topic that were expected after playing the game.

**Figure 2:**
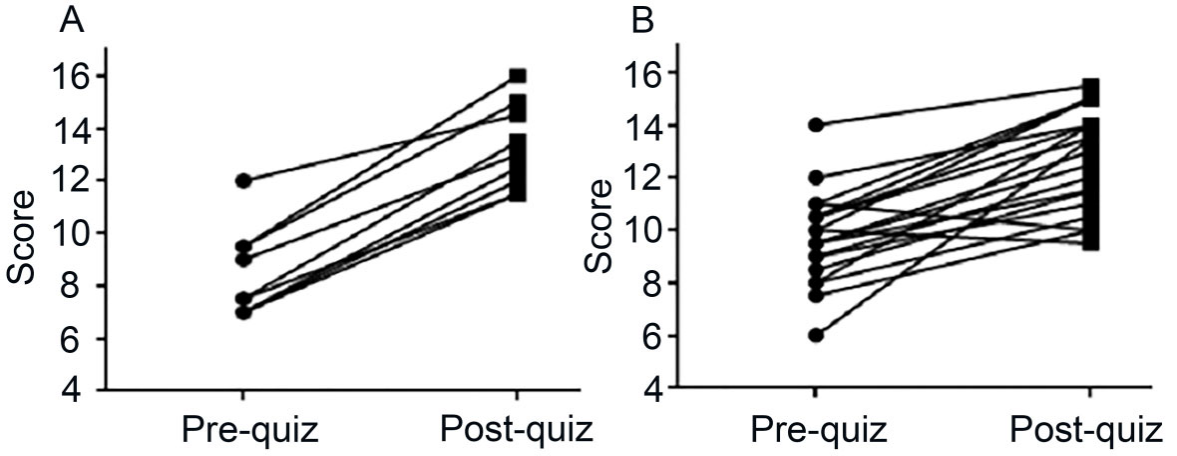
Assessment of learning after playing Screener in two courses. **A**, São Paulo State University “Júlio de Mesquita Filho” - UNESP (9 students): new drug discovery and development (DDD)-centered discipline with Screener played during three sessions (8 h of playtime). The post-quiz was administered immediately after the end of the game. Pre- and post-quiz scores (means±SD): 8.4±0.6 and 13.3±0.6, respectively (P<0.0001, paired *t*-test). **B**, Federal University of Ceará - UFC (19 students): existing discipline without DDD focus where Screener was played during one session (2.75 h of playtime). The post-quiz was conducted the day after the end of the game. Pre- and post-quiz scores (means±SD): 9.7±0.4 and 12.7±0.7, respectively (P<0.0001, paired *t*-test).

This hypothesis was confirmed when the quizzes were administered in two postgraduate courses that employed the game and applied the post-test in different ways (Figure 2). In both cases, the baseline level (pre-test) of postgraduate students was higher than that of undergraduate students. Nevertheless, the students' performance improved after playing the game, with scores increasing from 8.4 to 13.3 (P<0.0001, paired *t*-test) and 9.7 to 12.7 (P<0.0001, paired *t*-test).

### Qualitative assessment of the use of Screener as a teaching tool

Surprisingly, our game has been used in undergraduate courses such as Nursing at the Federal University of Mato Grosso (UFMT), Biotechnology at the School of Art, Sciences and Humanities of the University of São Paulo (EACH-USP), and Medicine and Physiotherapy at the Federal University of Espirito Santo (UFES). It has also been used in a technical course (Federal Institute of Rio de Janeiro, IFRJ), specifically in the last year of a pharmacy degree program. Some professors have creatively adapted the game to shorten its duration and focus on the main points. Despite the modifications, the use of Screener has been well-received by both students and teachers (personal communications).

### Qualitative assessment of Screener in new developments and collaborations

Among the eleven postgraduate courses that reported using Screener from June 2022 to June 2023, six courses created a specific discipline centered on the game, while five courses incorporated the game into the activities of an existing, more general discipline. Additionally, other courses requested and received the game, and more requests are expected as the information about Screener as an engaging and effective tool spreads within the network of SBFTE postgraduate members and beyond. Notably, Screener has been utilized in ten states of Brazil (postgraduate and undergraduate courses).

## Discussion

### Student perception

To quantitatively assess individual student perceptions across all postgraduate courses that used Screener in the classroom, we used a validated questionnaire (Meega+) that evaluates games in terms of quality factors and a set of nine dimensions. This approach is common in the Pharmacy area, where educational games are often evaluated through surveys, assessments, or student grades (2). Overall, the Screener game received highly positive evaluations, probably because it contains structural elements that are essential for an effective gamification tool, such as clear rules, competition, conflict, challenge, and story representation (2). However, student perception of the game rules was not as positive as expected, despite the detailed illustrated rule book (7). Considerable variation was observed in the results for this point across courses, which can be attributed to several factors such as the fact that not all programs did a formal presentation of the game and its rules before playing the game, as suggested by the game authors. Although categorized as a collaborative game, Screener also has elements of competition with a winner at the end of the game. The game also involves conflict, such as when a player must decide whether to use its power card, challenge another player to define a technical term, or determine the best strategy for being the first to take a task card. Throughout the game, players are constantly challenged as they must explain the content of all task cards, including the 59 technical terms present in some cards (7). Finally, the players construct the story of the DDD process as they align the task cards on the Process Map and need to provide brief summaries of the tasks performed after the completion of each of the seven stages. Most importantly, all students reported feeling a sense of achievement and overall satisfaction at the end of the game.

### Learning

According to Hope et al. (2), “learning is optimized by the presence of gamified mechanisms, such as immediate feedback, active participation, practice, motivation, and teamwork as these support a socio-constructivist pedagogy that is responsive to the needs of contemporary learners”. Screener fulfills these criteria in the following ways, providing an explanation for the very positive data on learning perception shown in Figure 1: first, students actively participate in the game as they are the players and have to explain all the information on the cards. The monitor only participates occasionally if some additional comments are relevant. Second, immediate feedback is provided each time a student picks up a task card, explains a bonus/setback card, or helps a colleague explain a topic. Third, the game includes suggestions in the rule book to help consolidate knowledge through repetition. For example, the player who buys the last task card of a stage should make comments about what was achieved in that stage by reading the name of the stage and the titles of the cards accumulated throughout the stage, placed on the process map. Fourth, motivation is achieved by the perceived relevance of the game, as evidenced by the positive responses to the relevance statements: “The content of the game is relevant to my interests”; “it is clear to me how the content of the game relates to the course”; and “this game is an adequate teaching method for this course”. Finally, Screener is a collaborative game that incorporates the essential aspect of teamwork that is crucial for any game. In this case, it particularly emphasizes the multidisciplinary aspect of DDD in real life.

However, as recommended in a recent systematic review (1), it is also important to directly measure learning outcomes related to specific knowledge acquisition in order to determine the actual benefits of using games. The authors pointed out that such assessments are not easy to conduct, which is why they are rarely reported. As an initial step towards achieving this objective, we developed a pre-post quiz and implemented it at two different locations. The results clearly showed that playing Screener significantly increased students' knowledge on DDD.

### Comparative evaluation

After comparing Screener with four other board and/or card games that use the DDD process (Supplementary Table S1), it can be concluded that Screener has several advantages over the others. These include its free availability, the inclusion of QR codes that provide in-depth technical information, and its modern/appealing design. Additionally, no trained instructor is required for playing the game. However, the restriction that the game is only available in Portuguese is a significant obstacle to wider international use of the game.

### Conclusion

The original educational board-game with online resources, Screener, designed to teach various aspects of the DDD process to postgraduate students, has received positive feedback when used in eleven courses. The results of a specialized and validated survey to assess the game's quality strongly indicate that Screener is a useful and enjoyable tool for promoting positive learning. This is further supported by a direct assessment of students' knowledge of DDD through pre- and post-quizzes. Additionally, implementation of Screener in Brazilian postgraduate courses articulated and supported by the Brazilian Society of Pharmacology and Experimental Therapeutics (SBFTE) was instrumental in the feasibility of this assessment. Of note, in a country of continental size such as Brazil, the use of Screener has the potential to decrease the inequalities in education between different regions, as demonstrated by our data.

## Supplementary Material

Click to view [pdf].
