# Supplementary material for: Assessing the reaction to and efficacy of the Screener drug discovery and development board game as a pedagogical tool in postgraduate courses
Source: Braz J Med Biol Res. 2024 Jan 22;57:e13258. doi: 10.1590/1414-431X2023e13258 (PMC10802222; doi:10.1590/1414-431X2023e13258)
Supplement: Supplementary file 1 [file 1414-431X-bjmbr-57-e13258-suppl.pdf]

Table S1. Board and/or card games on the drug discovery and development (DDD) process.

Name & description	Availability	Target public	Positive aspects	Limitations	Monitor	No. players (teams)	Playing time	Evaluation criteria
MeduMaZe, Board race game, 57 spaces.	2005–2012. No longer available.	Industry, academic, professional association.	Regulatory aspects, Bonus/setback, Glossary.	Little technical information on assays in the discovery stage.	Needs qualified monitor.	4–6	0.45–1 h	None
The PHARM game 2, Board race game, 72 spaces.	Available https://thelearningkey.square.site/	Corporate training.	Regulatory aspects.	Price: US$2,495.00	Unknown.	2–3	2.5–3 h	None
The Biotech game of life, Board race game, 72 spaces.	Available online https://www.scq.ubc.ca/the-biotech-game-of-life/	Undefined.	Free.	No technical information on the assays.	Not needed.	2–4	Unknown	None
Unnamed*. Card game.	Cards to be created by students.	Undergraduate students in pharmacy	Positive and negative cards. No cost.	Not a "ready to play" game. No glossary	Needs qualified monitor.	6	4 h	Pre- *vs* Post-test
SCREENER, Hybrid board and card game. Race game, 30 tiles.	Available on line: www.screener.com.br	Postgraduate students in pharmacology and related areas.	Free QR codes (cards). Bonus/setback cards. Glossary. Assays. 'Information' book.	Portuguese. No Regulatory aspects.	Not needed.	6	6–8 h	Validated survey

*Lounsbury N, Nicholas K, Chow NK, Jackson KC, Chbib C. Creation and implementation of a drug discovery and development game. *Curr Pharm Teach Learn* 2022; 14: 229–234, https://doi.org/10.1016/j.cptl.2021.11.025.
